# Changes in the Carbohydrate Profile in Common Buckwheat (*Fagopyrum esculentum* Moench) Seedlings Induced by Cold Stress and Dehydration

**DOI:** 10.3390/metabo13050672

**Published:** 2023-05-19

**Authors:** Lesław B. Lahuta, Ryszard J. Górecki, Joanna Szablińska-Piernik, Marcin Horbowicz

**Affiliations:** Department of Plant Physiology, Genetics and Biotechnology, University of Warmia and Mazury, Oczapowskiego 1a, 10-719 Olsztyn, Polandrigorecki@gmail.com (R.J.G.); joanna.szablinska@uwm.edu.pl (J.S.-P.)

**Keywords:** common buckwheat, seedling, cold stress, dehydration, carbohydrates, cyclitols, galactosides, raffinose, stachyose

## Abstract

Plant species are sensitive to stresses, especially at the seedling stage, and they respond to these conditions by making metabolic changes to counteract the negative effects of this. The objectives of this study were to determine carbohydrate profile in particular organs (roots, hypocotyl, and cotyledons) of common buckwheat seedlings and to verify whether carbohydrate accumulation is similar or not in the organs in response to cold stress and dehydration. Roots, hypocotyl, and cotyledons of common buckwheat seedlings have various saccharide compositions. The highest concentrations of cyclitols, raffinose, and stachyose were found in the hypocotyl, indicating that they may be transported from cotyledons, although this needs further studies. Accumulation of raffinose and stachyose is a strong indicator of the response of all buckwheat organs to introduced cold stress. Besides, cold conditions reduced d-*chiro*-inositol content, but did not affect d-pinitol level. Enhanced accumulation of raffinose and stachyose were also a distinct response of all organs against dehydration at ambient temperature. The process causes also a large decrease in the content of d-pinitol in buckwheat hypocotyl, which may indicate its transformation to d-*chiro*-inositol whose content increased at that time. In general, the sucrose and its galactosides in hypocotyl tissues were subject to the highest changes to the applied cold and dehydration conditions compared to the cotyledons and roots. This may indicate tissue differences in the functioning of the protective system(s) against such threats.

## 1. Introduction

Common buckwheat (*Fagopyrum esculentum* Moench) is a species native to the cool, foothill regions of Northeast Asia [1]. Buckwheat seeds contain valuable protein due to their balanced composition and high lysine content [2,3]. Buckwheat also contains flavonoids, and the lack of gluten proteins distinguishes buckwheat from commonly consumed cereal products [4,5]. Besides starch and sucrose, buckwheat seeds also contain specific saccharides, d-*chiro*-inositol (DCI) and its galactosides, called fagopyritols [6,7,8,9]. The accumulation of DCI and fagopyritols in buckwheat tissues has led to increased interest in this species for dietary and medical reasons [10]. DCI and its galactosamine derivative is a putative insulin mediator, useful in the treatment of type 2 diabetes [11,12]. Besides, fagopyritol A1 is structurally similar to the galactosamine derivative of DCI [13]. Polycystic ovary syndrome (PCOS) is one of the most common causes of menstrual irregularity during which women have lower plasma DCI levels [14]. Therefore, DCI and fagopyritol A1 may also have applications in the treatment of patients with PCOS [15]. DCI and d-pinitol (PIN) glycans have also been shown to have some therapeutic potential in Alzheimer’s disease [16]. Buckwheat seeds are also used to prepare sprouts and microgreens, which are widely regarded as a rich source of bioactive compounds, such as flavonoids, DCI, and its galactosides [17]. On the other hand, *myo*-Inositol (MIN) and DCI are osmolytes that protect plant tissues from stress or metabolic disorders [18,19]. Both inositols are involved in the synthesis of glycosyl-phosphatidyl-inositol and inositol phosphoglycans [20]. 

Drought stress during plant vegetation limits plant development and crop productivity [21]. Plants have evolved specific molecular, cellular, and physiological response mechanisms to drought stress conditions [22]. The well-known accumulation of osmolytes under drought helps maintain osmotic balance under dehydration [23]. Osmolytes include some carbohydrates, proline, quaternary ammonium compounds, and tertiary sulfonium compounds [24]. A common event under dehydration of plant tissue is the accumulation of soluble carbohydrates, such as the raffinose family oligosaccharides (RFOs) for protection and stabilizing of proteins [25,26]. However, water deficiency during vegetation of some tree species causes a reduction of most sugars, organic acids, and phenolic compounds [27].

Accumulation of mono- and oligosaccharides has been demonstrated in response to drought stress in many tropical plant species [28]. Under such stress, Arabidopsis plants accumulate large amounts of raffinose and galactinol, whereas these carbohydrates were not detected in non-stressed plants [29]. These results show that galactosides may function as osmoprotectants in the stress tolerance of plants. 

Cyclitols and/or their galactosides accumulate in response to drought stress [30,31,32]. According to Liu and Grieve [33], cyclitols accumulation may be a species-characteristic physiological feature for halophytic *Limonium* species. The authors found that DCI and MIN are transported through the phloem from the leaves to the roots. They suggest that the flow of cyclitols through the phloem appears to be important as it was intensified by salt stress. In addition, significantly more DCI was exuded from the roots than MIN. Sucrose and DCI were the main carbohydrates in the sap exuded from the phloem. Cyclitols play an important role in the life of living organisms participating in many biological processes as evidenced by a large number of scientific reports and reviews concerning them [34].

During vegetation under field conditions, plants are exposed to cold stress from germination through seedling growth to seed release. Buckwheat is a species sensitive to abiotic stresses, especially at the seedling stage [35]. In our previous study, it was shown that during buckwheat field production the air temperature can vary from −0.5 to 31 °C and the soil temperature from 4.5 to 21 °C [36]. Plants respond to these conditions by making metabolic changes to counteract the negative effects of this stress, and saccharides play an important role in the response to low-temperature conditions [37,38,39]. Increases in mono- and oligosaccharides have been shown to be common metabolic responses to that type of stress [32,40,41]. Also cyclitols (PIN, MIN, and ononitol) and α-galactosides of sucrose (RFOs) are involved in osmotic regulation in the response of a plant to environmental stress [42]. For instance, an increase in RFO content was found in rice seedlings grown in low temperatures [43]. Based on metabolic profiling, it was shown that the contents of most soluble carbohydrates significantly increase in tartary buckwheat tissues in response to cold stress [44]. Moreover, previously it was shown in buckwheat seeds that the accumulation of DCI and its mono galactosides (fagopyritol B1 and fagopyritol A1) is preferred at low temperature (15 °C) during seed maturation, whereas the accumulation of DCI di-galactosides (fagopyritol B2 and fagopyritol A2) is favored at high temperature (30 °C) [7]. 

Cold weather during plant vegetation affects many physiological and biochemical processes. The process of photosynthesis and the biosynthesis of the saccharides that are its products were particularly affected by cold stress during the growth of *Camellia* seedlings [45]. This was associated with a decrease in chlorophyll fluorescence and other photosynthetic parameters. This resulted in an increased content of UDP-glucose, UDP-d-apiose and fructose-6-phosphate, which, presumably, may contribute to increased cold tolerance in these plants. Also, the efficiency of both photosystems declined in cold-stressed plants of *Stevia rebaudiana*, which was accompanied by reductions of net photosynthesis, as well as of chlorophylls and carotenoid contents [46]. Besides, the cold stress increased water-soluble carbohydrates. 

The objectives of this study were to verify whether carbohydrate accumulation is similar in the organs of common buckwheat seedlings in response to cold stress and dehydration and if the application of cold stress or dehydration at ambient temperature can be used to enhance the content of pharmacologically important cyclitols in seedlings of common buckwheat. Since cold weather is common in Poland’s main buckwheat cultivation region, assessing how it affects primary metabolites of buckwheat plants seems important. Therefore, the following hypothesis was formulated: the introduction of cold conditions or dehydration after favorable conditions affect the profile and metabolism of carbohydrates in the roots, hypocotyl, and cotyledons of buckwheat seedlings.

## 2. Materials and Methods

Seedlings of common buckwheat (*Fagopyrum esculentum* Moench) cv. Kora were used in this study. Buckwheat seeds came from the online garden store Sadowniczy.pl. Germination was carried out by placing buckwheat seeds (20 in each of six replicates) between two layers of wet germination paper (Eurochem BGD, Tarnów, Poland) which were then rolled up and inserted into a 250 mL beaker containing 50 mL tap water. Beakers were kept for seven days in a growth chamber (EKO-POL ILW 115-T STD, Wodzisław Śląski, Poland) in 14/10 h photoperiod and 65 ± 5% relative humidity. Chamber temperature was maintained at 20 ± 1 °C/10 ± 1 °C (day/night). Light was provided by three daylight LED cold fluorescent lamps (330 lm/m^2^).

Seven-day-old buckwheat seedlings (ca. 200) were dehydrated with free air circulation under laboratory conditions (23 ± 2 °C, 22% RH) for 14 days under continuous light conditions. After this process, the seedlings were divided into roots, hypocotyls, and cotyledons in which dry-weight measurements and saccharide profile analyses were performed separately. Another set (ca. 200) intact buckwheat seedlings was subjected to seven days of cold stress in the growth chamber in 14/10 h photoperiod and temperatures of 5 °C during the day and 0 °C at night. The results were not compared to seedlings growing under favorable conditions without cold (20 ± 1 °C/10 ± 1 °C, day/night).

After the experiments were completed, each organ of buckwheat seedlings was weighed and, after freezing with liquid nitrogen, stored in a freezer at −75 °C. Before analyses, samples were freeze-dried for 48 h (freeze-dryer Alpha 1-2 LD plus, Martin Christ, Osterode, Germany). The dry material was ground in a vibratory mill (MM200, Retsch, Verder Group, Utrecht, The Netherlands).

### 2.1. Analyses of Soluble Carbohydrates

Soluble carbohydrates were extracted from 40 to 45 mg of pulverized samples with 800 μL of ethanol: water (1:1, *v/v*, at 90 °C for 30 min with continuous shaking at 300 rpm), containing 100 μg of xylitol (internal standard). Homogenates were centrifuged (20,000× *g* at 4 °C for 20 min) and aliquots (400 μL) of clear supernatants were deionized (with a 300 μL of a mixture of Dowex^®^ ion-exchange resins (Dowex 1X8 Cl^−^ form, 50–100 mesh, and Dowex 50WX8 H^+^ form, 100–200 mesh, cat. no. 217417 and 217492, respectively, Sigma-Aldrich, St. Louis, MO, USA) and brought to dryness in a speed-vacuum rotary evaporator. The dry residues were derivatized with a 200 μL of mixture of TMSI/pyridine (1:1, *v*/*v*, Sigma-Aldrich, St. Louis, MO, USA) at 80 °C for 45 min. The TMS derivatives of soluble carbohydrates were analyzed with the high-resolution gas chromatography method on a capillary column (Rtx-1, 15 m length, 0.25 mm diameter, 0.1 μm thickness of 100% dimethyl polysiloxane layer, Restek, Anchem Plus, Warsaw, Poland) in a gas chromatograph (GC2010, Shimadzu, Kyoto, Japan), under conditions described previously [47]. The results of the analyses were achieved using the internal standard method of calculation. Standards of xylitol, d-pinitol, d-*chiro*-inositol, and *myo*-inositol were purchased from Sigma-Aldrich (St. Louis, MO, USA). Standard galactinol was supplied by Wako Pure Chemicals Industries Ltd. (Osaka, Japan). Galactosides of d-pinitol (galactosyl pinitol A and B and di-galactosyl pinitol A—ciceritol), were extracted from seeds of winter vetch *(Vicia villosa* Roth) and purified as described previously [48]. Fagopyritols A1, B1, A2, and B2 were isolated and purified from seeds of common buckwheat [6,47,48,49,50,51,52]. 

### 2.2. Statistics

Analysis of variance and Tukey’s post hoc test were used to check the significance of differences. Results presented as means of three biological replicates marked with the same letter were not significantly different at *p* ≤ 0.05 (Tukey’s post hoc test). 

## 3. Results and Discussion

### 3.1. Carbohydrate Profile in the Organs of Buckwheat Seedling

**Figure 1:**
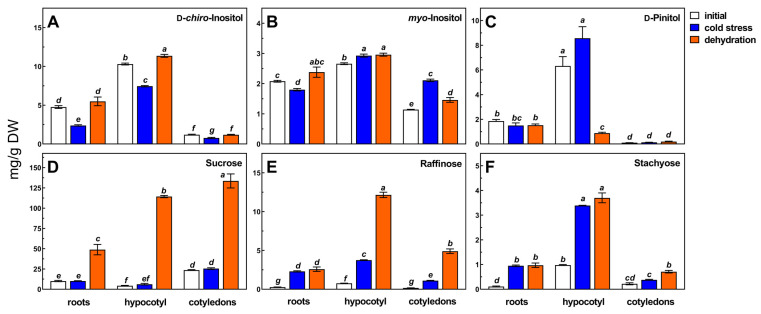
The content of cyclitols (**A**–**C**), sucrose (**D**), raffinose (**E**), and stachyose (**F**) in roots, hypocotyl, and cotyledons of seven-day-old buckwheat seedlings before stress (initial), and after seven days of cold stress or 14 days of dehydration at ambient temperature. Results presented as means marked with the same letter were not significantly different at *p* ≤ 0.05 (Tukey’s post hoc test).

**Figure 2:**
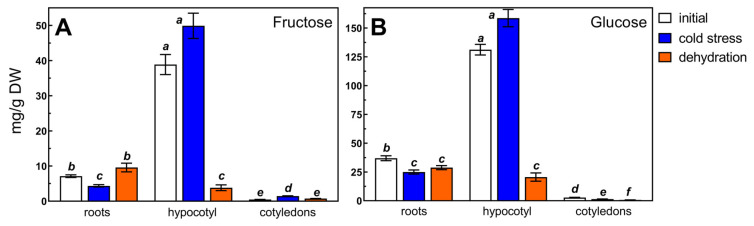
The content of fructose (**A**) and glucose (**B**) in roots, hypocotyl, and cotyledons of seven-day-old buckwheat seedlings before stress (initial), and after seven days of cold stress or 14 days of dehydration at ambient temperature. Results presented as means marked with the same letter were not significantly different at *p* ≤ 0.05 (Tukey’s post hoc test).

High-resolution gas chromatography analyses showed that in seedling organs of common buckwheat 15 soluble saccharides were presented, including cyclitols (*myo*-inositol, d-*chiro*-inositol, and d-pinitol), sucrose, raffinose, and stachyose (Figure 1), monosaccharides (glucose and fructose, Figure 2), maltose, and α-d-galactosides of cyclitols (Table 1, Figure S1).

These saccharides were identified based on the retention time of commercially available standards and those prepared from plant sources [5,6,46,47,48,49]. Among them, cyclitols (DCI, PIN, and MIN) were present in all organs, as well as monosaccharides (glucose and fructose), disaccharides (sucrose and maltose), and α-d-galactosides of sucrose (raffinose and stachyose). On the other hand, mono- and di-α-d-galactosides of DCI (fagopyritol A1, fagopyritol A2, and fagopyritol B1), and mono-α-d-galactosides of MIN (galactinol) and PIN (galacto-pinitol A and galacto-pinitol B) were mainly found in cotyledons, although fagopyritol B1 and galacto-pinitol A were present in all examined organs (Table 1). The chemical names and structures of cyclitols and their α-d-galactosides are shown in Figure S1. Among those listed, sucrose, glucose, and fructose were quantitatively dominant saccharides in all organs. Next in terms of content were DCI and PIN. The highest contents of the mentioned saccharides occurred in hypocotyl, much lower in roots, and the lowest in cotyledons (Figure 1 and Figure 2, Table 1). In addition to the saccharides identified by comparison to the retention time of their pure standards, several unidentified compounds (NN1–NN5) were present on chromatograms (Figure S2). The retention times of two of them (NN4 and NN5) were between raffinose and stachyose. Their contents were calculated based on the peak areas of the ciceritol (di-galactosyl pinitol A) standard (Table 1).

Seeds of common buckwheat *(Fagopyrum* esculetnum Moench) are a known plant source of DCI and a set of six fagopyritols, which are mono-galactosides of the DCI (fagopyritol A1 and fagopyritol B1), di-galactosides (fagopyritol A2 and fagopyritol B2), as well as tri-galactosides (fagopyritol A3 and fagopyritol B3) [6,7,9,15,49,51]. The presence of three of them, i.e., fagopyritol A1, fagopyritol A2, and fagopyritol B1, was confirmed in seedling organs of common buckwheat (Table 1). However, their contents were low or below the limit of quantification. This is explicable because fagopyritols are degraded during seed germination and growth of buckwheat seedlings, as was earlier reported [53]. The presence in the analyzed tissues of two isomeric PIN galactosides, i.e., galacto-pinitol A and galacto-pinitol B, was shown for a first time. The significantly higher content of galacto-pinitol A in hypocotyl and roots than in cotyledons may suggest that this saccharide may be transported from leaves to roots. However, this requires further confirmation. Much higher content of cyclitols and RFOs in hypocotyl tissue than in cotyledons is probably due to the fact that they are transported by phloem, as found in many tree species (Figure 1) [54,55]. On the other hand, plants can maintain high concentrations of RFO and galactinol in the phloem by producing them in intermediate cells, as reported in the *Cucurbitaceae* family [56]. In contrast, the accumulation of sucrose, raffinose, and stachyose was reduced while transport outside the leaf was efficient. In *Limonium* species DCI and MIN are transported through the phloem from leaves to roots [33]. The authors have shown that sucrose and DCI were the predominant carbohydrates in phloem-exuded sap, and their excretion from the roots to soil plays specific roles in plant function and their interaction with the environment [56,57].

Besides, the chromatograms showed peaks of unknown compounds between the peaks of raffinose and stachyose. Presumably, one of them is di-galacto-pinitol B, which is an isomer of known ciceritol (di-galacto-pinitol A). The other is probably di-galacto-*myo*-inositol (DGMI) or tri-galacto-pinitol. There are no data on their presence in buckwheat tissues in the previously published papers. 

Plants rich in PIN are being used in traditional medicine as a treatment for diabetes, inflammation, or other diseases [58]. Earlier, it was found that PIN administration lowered blood sugar levels in diabetic albino mice [59]. However, PIN by itself does not exhibit antidiabetic effects but DCI does. Therefore, PIN is presumed to undergo enzymatic demethylation in mammalian cells, and the resulting DCI is an insulin mediator and lowers plasma glucose concentrations. Suggestions on biosynthesis of DCI in plants through demethylation of PIN has been published many times although the enzyme responsible for this has not been described [60,61,62,63]. It seems that content DCI is more important than PIN for drug-related reasons. Medical research has shown that DCI and its derivative galactosamine is a putative insulin mediator useful in the treatment of type 2 diabetes [11,12,13]. Moreover, a menstrual irregularity called polycystic ovary syndrome (PCOS) has been observed in women who have low plasma DCI levels [14,15]. DCI and its glycans have also been shown to have some therapeutic potential in treating Alzheimer’s disease [16]. All these facts show DCI and PIN as important components in the human diet, and knowing how they behave in plant tissues is essential.

### 3.2. Cold Stress and Carbohydrate Profile in Organs of Common Buckwheat Seedlings

Cold stress caused a decrease in DCI in all tested organs of buckwheat seedlings (Figure 1A). The stress decreased MIN in roots but increased it in hypocotyl and cotyledons (Figure 1B). In contrast, cold stress did not affect PIN content in all organs of buckwheat seedlings (Figure 1C). Raffinose was the saccharide whose content was most affected by cold stress (Figure 1E). The stress increased its content 8-fold in roots, 5-fold in hypocotyl, and 6.5-fold in cotyledons of buckwheat seedlings. The cold stress also enhanced stachyose content 8-fold in roots, but only 3.5-fold in hypocotyl, and about 2-fold in cotyledons (Figure 1F). Cold stress had the least effect on sucrose content, which did not increase in roots and cotyledons after its influence, while its increase in hypocotyl was about 40% (Figure 1D). 

Similar effects of cold stress on PIN and DCI contents in hypocotyl, roots, and cotyledons of buckwheat seedlings (Figure 1A,C) may confirm a metabolic link between both cyclitols. In previous papers, it was suggested that the presence of PIN in buckwheat tissues may be due to the involvement of this cyclitol as an intermediate in the biosynthesis of DCI from MIN [7,8]. The reduction in root and hypocotyl DCI content as a result of cold stress may have been related to an increase in the content of one of its mono-galactosides, fagopyritol B1 (Table 1). There was also a different tissue response to cold stress in MIN, i.e., a decrease in the roots and an increase in the hypocotyl and cotyledons. Changes in the concentration of MIN can be associated with its use in the synthesis of galactinol, a compound that increased slightly during cold stress in cotyledons and hypocotyls. Besides, cold stress did not affect sucrose levels but markedly increased raffinose and stachyose contents in all organs of buckwheat seedlings (Figure 1E,F).

The applied cold conditions caused a slight decrease in the content of fructose and glucose in the roots, their slight elevation in the cotyledons, and a non-significant increase in the hypocotyl (Figure 2). The cold stress also did not affect the concentrations of maltose in the organs evaluated and the contents of DCI mono-galactosides (fagopyritol A1 and B1) in cotyledons, but slightly raised the content of MIN galactoside (galactinol) in cotyledons and hypocotyl (Table 1). In turn, the level of fagopyritol B1 was enhanced in cold-stressed tissues of hypocotyl and roots. 

The cold stress reduced the fructose and glucose content in roots, as well as the glucose content in cotyledons, which may indicate increased utilization in enhanced respiratory processes. The cold factor, on the other hand, caused an increase in fructose levels in cotyledons and the contents of galacto-pinitol A, galacto-pinitol B, and fagopyritol B1 in roots. The different metabolic responses in buckwheat organs to cold stress may indicate various defense mechanisms in a particular organ by biosynthesis of additional defense metabolites, such as proline, or another metabolic response. In buckwheat seeds, the relatively low temperature during seed maturation resulted in an increase in fagopyritol A1 and fagopyritol B1, compared to the higher temperatures [7]. An increase in fagopyritol B1 content was also found in the cold-stressed roots and hypocotyl of buckwheat seedlings. However, the substantially higher increase in raffinose and stachyose content in all organs of buckwheat seedlings indicates that RFOs are cold-stress protectants in seedlings of this species.

The phenomenon of saccharide accumulation under cold stress has been described many times [64,65,66]. In Arabidopsis leaves, an increase in transcription of three genes encoding galactinol synthase, an enzyme important in the synthesis of raffinose, has been demonstrated in response to low temperatures [65]. In three kale types exposed to low temperatures, maltose and sucrose decreased while the concentrations of fructose, melibiose, maltose, raffinose, and galactinol were increased [67]. However, the full mechanism of metabolic response to cold stress seems to be much more complicated than the accumulation of carbohydrates. According to Fowler and Thomashow [66], the expression of more than 300 genes in Arabidopsis was affected by low temperature, with transcripts for 218 genes increasing and those for 88 genes decreasing 3-fold or more. Therefore, further research is needed here, especially on the transcription of genes related to this stress factor.

Marked increase in the dry weight of the hypocotyl of buckwheat seedling (by ca 25%) and its decrease in the cotyledons and roots after chilling period was noted (Table S1). Besides, during such growth conditions, the hypocotyl continued to grow, as it was about 37% longer after a seven-day period than at the beginning of the experiment. This indicates that the applied temperature (5 °C/0 °C days/nights) had not inhibited the photosynthesis in cotyledons and transport of assimilates to the hypocotyl.

### 3.3. Dehydration and Carbohydrate Profile in Organs of Common Buckwheat Seedling

Saccharides play an important role in protecting the cytoplasm during the desiccation of plant tissues [68]. Desiccation, or osmotic stress, induces accumulation of RFOs as well as expression of galactinol synthase and accumulation of galactinol [27,29,69,70,71]. The RFOs as well as the cyclitol galactosides provide osmotic regulation and protect the macromolecules and membranes [32]. Galactinol is the galactosyl donor for the synthesis of raffinose and stachyose by raffinose synthase and stachyose synthase, respectively [72,73]. In the present study, a simple process was used to dehydrate buckwheat organs by gradually keeping them for two weeks of intact seedlings at ambient temperature. As a result of this process, sucrose, raffinose, and stachyose contents were significantly more affected than those subjected to the cold stress (Figure 1C,D). In the hypocotyl, the sucrose content after dehydration was about 26-fold higher compared to the content before the process, and in roots and cotyledons by 5-fold and 6-fold, respectively. Probably invertase activity decreased rapidly during water loss, and this inhibited hydrolysis of sucrose. The accumulation of sucrose in cotyledons to a concentration higher than that in hypocotyl and root seems to depend on the rate of dehydration of each seedling’s part. Roots dehydrated first, hypocotyl a few days later, while in cotyledons, the water content below 6% of the fresh weight was noted after 12–14 days of dehydration (data not shown). Nonetheless, the accumulation of sucrose in each seedling’s part (Figure 1D), additionally accompanied by a decrease in monosaccharides in hypocotyl (Figure 2), is a confirmation of the participation of sucrose in cells’ osmotic adjustment during dehydration and/or sucrose osmoprotective role under water stress [74].

Besides sucrose, raffinose was the second most responsive saccharide to dehydration, as its increase was 10-fold in roots, 16-fold in hypocotyl, and almost 29-fold in cotyledons (Figure 1E). Moreover, the content of stachyose after dehydration increased almost 9-fold in roots, 4-fold in hypocotyl, and more than 3-fold in cotyledons of buckwheat seedlings (Figure 1F). Probably raffinose and stachyose synthases are active for a long time during the dehydration process resulting in a gradual accumulation of both galactosides. The observed multiple increases in sucrose, raffinose, and stachyose confirm many previous reports showing the role of these saccharides during dehydration [32,68,75] or drought stress conditions [27,29,69,70,71]. Although the concentration of sucrose after dehydration was much higher than that of raffinose and stachyose (regardless of seedling organs) the protective role of RFOs under desiccation seems to be the prevention of sucrose crystallization (maintaining a glassy state of cytoplasm) under extremely low water content and stabilization of structure of membranes and macromolecules [32,76]. 

The changes in cyclitols during dehydration are less known. The dehydration at ambient temperature increased MIN content in all organs of buckwheat seedlings, while DCI increased in roots and hypocotyls (Figure 1A). The process, furthermore, dramatically reduced the PIN content in the hypocotyl, without affecting its levels in cotyledons and roots (Figure 1C).

Dehydration repeatedly lowered the fructose and glucose contents of the hypocotyl without affecting their content in cotyledons and roots (Figure 2). It also slightly increased the contents of maltose in roots and hypocotyl and fagopyritol B1 in the hypocotyl (Table 1). In some plant species, the role of cyclitol (mainly DCI) galactosides is dependent on the organ undergoing a process of dehydration. Such a species is buckwheat, in which fagopyritol B1 (mono-galactoside of DCI) is accumulated on intact plants during seed desiccation [6]. In contrast, after the desiccation of buckwheat seedlings for seven days at 22 °C, raffinose and stachyose accumulated in hypocotyl tissues instead of DCI galactosides [53]. Therefore, the aim of our present study was to verify whether the response of particular organs of buckwheat seedlings to desiccation is the same or different. 

The concentration of galactinol in roots and cotyledons of buckwheat seedlings was below the limit of determination. This was also accompanied by a response in the accumulation of the content of DCI and an elevation of its galactoside (fagopyritol B1) in the hypocotyl. In addition, the content of PIN was then drastically reduced, and MIN content was slightly enhanced. In other plants, a high increase in PIN accumulation in response to drought stress was found [77,78,79,80,81,82]. High PIN accumulation correlated with decreased content of its direct precursor MIN, and was found in the leaves of pigeon pea (*Cajanus cajan*) plants subjected to drought stress [78]. Also, Orthen et al. [80] have shown that PIN was accumulated in the leaves, shoot axis, and roots of intact chickpea (Cicer arietinum) plants in response to drought stress. In this species, 14 days of water stress increased PIN content from 5 to 195 mmol/kg DW in the leaves, from 9 to 67 mmol/kg DW in the shoot axis, and from 5 to 22 mmol/kg DW in the roots. Later, Streeter et al. [81] found that the molar concentration of PIN in leaf blades of soybean plants subjected to drought was much higher than the concentrations of proline or sugars. Recently, it was shown that water deficit induced an increase in PIN concentration in all soybean organs and lowered concentrations of MIN in leaf samples [82]. Dehydration of buckwheat hypocotyl causing a marked decrease in PIN content which may indicate that this cyclitol is broken down into MIN. The decrease in PIN in the hypocotyl (Figure 1) can also be partially explained by an increase in galactopinitol A content (Table 1). A similar increase in this galactoside occurred in dehydrated roots of buckwheat seedlings. 

The large reduction in PIN content in buckwheat hypocotyl after the dehydration of seedlings under ambient conditions is the opposite of that observed in four-week-old buckwheat seedlings after drought stress induced by the application of 10% PEG-6000 solutions [83]. After three days of such stress, the contents of DCI, PIN, and MIN were many-fold higher than in the unstressed control. However, our buckwheat plants were much younger and the dehydration is not the same as the treatment of intact seedlings by PEG [83].

In the root and cotyledon tissues of buckwheat seedlings, the dehydration process did not affect the contents of DCI, MIN, and PIN. There was only a slight increase in fagopyritol B1 in the seedling roots. These all data indicate that galactosides of cyclitols are less important in defense mechanisms in response to the desiccation stress in organs of common buckwheat seedlings. This is different from the phenomenon demonstrated during the natural seed desiccation process on intact plants of common buckwheat [6].

An increase in MIN content in buckwheat hypocotyl with a large decrease in PIN may indicate an unknown metabolic pathway in buckwheat than that demonstrated in legumes [82]. The precursor in PIN synthesis is MIN and the intermediate metabolite is ononitol [77]. In this synthesis, the methyl donor is S-adenosyl methionine and the enzyme is inositol methyl transferase, whose activity leads to the synthesis of ononitol (d-4-*O*-methyl-*myo*-inositol) [77]. Both enzyme and mRNA transcript levels were elevated in response to drought stress in pigeon pea (*Cajanus cajan*) [78]. A marked decrease in PIN content in the hypocotyl, found in our study, may suggest a process of its demethylation which may confirm an increase in DCI content. Although the biosynthesis of DCI in some plants seems possible through demethylation of PIN, the enzyme responsible for this has not been identified [60,61,62,63,84].

Galactinol is the galactosyl donor for the biosynthesis of both the RFOs and galactosyl cyclitols, and the enzyme in the pathway of RFOs and cyclitol galactosides biosynthesis is galactinol synthase [71,85]. Its expression under drought stress is well documented [29,69,70]. Galactinol synthase also catalyzes the synthesis of DCI galactosides (fagopyritol A1 and fagopyritol B1) [86]. Indeed, fagopyritol B1 content was 4-fold higher in dehydrated buckwheat hypocotyl, and 2-fold higher in roots than in their tissues before dehydration (Table 1). This confirms that dehydration of buckwheat seedlings increases galactinol synthase activity, although galactinol content was very low. Fagopyritol A1 was not found in measurable content in buckwheat roots and hypocotyl, and in dehydrated cotyledons its content was below the detection limit. 

**Figure 3:**
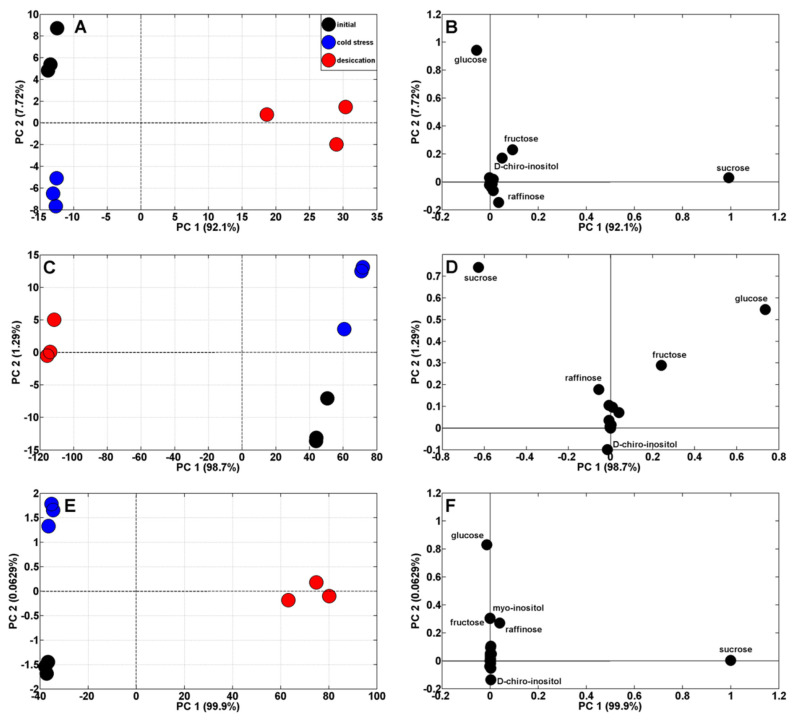
Principal component analysis (PCA) profiles of root (**A**), hypocotyl (**C**), and cotyledons (**E**) soluble carbohydrates in seedlings of buckwheat before (initial, black dots) and after cold stress (blue dots) and dehydration (red dots). PCA loading plots of soluble carbohydrates are shown on (**B**,**D**,**F**) for roots, hypocotyl, and cotyledons, respectively.

Principal components analysis (PCA) of soluble carbohydrates in buckwheat seedlings showed a clear separation of initial (control) and cold-stressed samples from those desiccated (Figure 3A,C,E).

The analytical results of control and cold-stressed roots and cotyledons were to the left of PC1, sharing 92.1 and 99.9% of the variability, whereas root and cotyledons samples of dehydrated seedlings were to the right (Figure 3A,E). The opposite distribution was found in samples of hypocotyls (Figure 3C). Moreover, the initial and cold-stressed samples of all seedling organs were also separated according to PC2, sharing only 0.06–7.72% of the variability. The major carbohydrates reflected in roots and cotyledons samples separation were sucrose and glucose in (Figure 3B,F), while the separation of hypocotyl samples was associated with the concentrations of sucrose, glucose, fructose, and raffinose (Figure 3D). 

The genus *Fagopyrum* includes two important food plants, such as *F. esculentum* (common buckwheat) and *F. tataricum* (Tartary buckwheat) [1,2,3]. The seeds, sprouts and seedlings of both types of buckwheat contain a whole set of phenolic compounds, such as phenolic acids, flavonols, flavones, catechins, and anthocyanins [85,86]. Therefore, buckwheat seedlings are convenient objects for examining the impacts of environmental factors on these compounds [87,88,89,90,91].

Several interesting reports on cold stress in Tartary buckwheat have been published recently [92,93,94]. Transcriptomic and metabolomic analyses showed that phenylpropanoid biosynthesis was elevated after cold treatment of seedlings, confirming that flavonoids play an important role in enhancing resistance to this stress [92]. The authors identified 24 genes involved in the synthesis of flavonoids in response to cold. Another study showed that in *F. tataricum*, low temperature caused significant changes in the expression of histone deacetylases (HDACs) which correlated with flavonoid synthesis pathway genes [93]. Besides, when *F. tataricum* was subjected to cold stress, the anthocyanin content in the epidermis and cortex cells of the hypocotyl was twice as high as in the cotyledons increases their resistance to cold [91]. Moreover, metabolic profiling based on GC-TOF-MS analysis showed that most sugars and their derivatives increase significantly in response to cold [94]. Also, DNA methylation may act as a regulator of the cold response in *F. tataricum* [94]. Application of a DNA methylation inhibitor altered free lysine content, indicating that DNA methylation affects metabolite accumulation in response to cold stress [94]. These data, and those obtained in our study, indicate the complexity of the response and process of cold tolerance in plants of the genus *Fagopyrum*.

## 4. Conclusions

The roots, hypocotyl, and cotyledons of common buckwheat seedlings have different saccharide profiles. The highest contents of total saccharides occurred in the hypocotyl, were lower in the roots, and lowest in their cotyledons. Also, in the hypocotyl there were the highest contents of all cyclitols as well as raffinose and stachyose, indicating that they may be transported. However, it needs confirmation in further studies.

Accumulation of raffinose and stachyose is a strong and clear indicator of the response of buckwheat tissues to cold stress. The applied cold-stress conditions resulted in lower d-*chiro*-inositol content and had no effect on d-pinitol content in all organs of buckwheat seedlings. However, these results have not been compared with those that could be obtained under favorable conditions, which may cause some doubt. The increase in hypocotyl dry weight observed may indicate the continuation of assimilate transport from to the hypocotyl under cold conditions. 

Accumulation of raffinose and stachyose is also a clear response of all organs of buckwheat seedlings to water loss during dehydration at ambient temperature. The process causes also a large decrease in the content of d-pinitol in buckwheat hypocotyl while d-*chiro*-inositol content increased at that time. The large decrease in fructose, glucose, and d-pinitol content in the hypocotyl during dehydration indicates that metabolic processes using these saccharides are then taking place. The accumulation of sucrose in each part of the buckwheat seedling confirms the contribution of this carbohydrate to the osmotic regulation of cells during dehydration and/or the osmoprotective role of sucrose in water stress.

The hypocotyl tissues showed the greatest changes in carbohydrate composition in response to cold and dehydration stress conditions, while those in cotyledons and roots were milder. This may indicate tissue differences in the functioning of the protective system(s) against such threats. Since the hypocotyl is the main organ by weight of buckwheat seedlings, changes in the content of pharmacologically important saccharides appear to be significant in it. Cold stress slightly reduced the d-*chiro*-inositol content of the hypocotyl but the level of total cyclitols remained unchanged. In contrast, dehydration of buckwheat organs caused a slight increase in the content of d-*chiro*-inositol but a large decrease in d-pinitol and total cyclitols.

## Figures and Tables

**Table 1:** Changes in the content of some carbohydrates in the organs of buckwheat seedlings before and after cold stress and dehydration at ambient temperature. Values (mg/g DW) are means ± SD.

Carbohydrate	Initial Tissue	Tissue after Cold Stress	Tissue after Dehydration
Roots			
Maltose	0.09 ± 0.04 ^b^	0.07 ± 0.01 ^b^	0.23 ± 0.02 ^a^
Galacto-Pinitol A	0.44 ± 0.11 ^a^	0.62 ± 0.04 ^a^	0.80 ± 0.07 ^a^
Galacto-Pinitol B	Nd *	0.14 ± 0.02 ^a^	0.10 ± 0.02 ^a^
Fagopyritol A1	Nd *	Nd *	Nd *
Fagopyritol A2	Nd *	Nd *	Nd *
Fagopyritol B1	0.09 ± 0.01 ^b^	0.24 ± 0.01 ^a^	0.18 ± 0.01 ^a^
Galactinol	Nd *	Nd *	Nd *
NN4	Nd *	0.23 ± 0.02 ^a^	0.08 ± 0.02 ^b^
NN5	Nd *	Nd *	Nd *
Total cyclitols	8.70 ± 0.29 ^a^	5.70 ± 0.19 ^b^	9.39 ± 0.80 ^a^
Total galactosyl cyclitols	0.53 ± 0.10 ^b^	1.00 ± 0.04 ^a^	1.09 ± 0.09 ^a^
Hypocotyl			
Maltose	0.10 ± 0.04 ^b^	0.09 ± 0.01 ^b^	0.57 ± 0.13 ^a^
Galacto-Pinitol A	0.22 ± 0.04 ^b^	0.36 ± 0.02 ^b^	0.65 ± 0.05 ^a^
Galacto-Pinitol B	0.11 ± 0.01 ^b^	0.49 ± 0.02 ^a^	Nd *
Fagopyritol A1	Nd *	Nd *	Nd *
Fagopyritol A2	Nd *	Nd *	Nd *
Fagopyritol B1	0.12 ± 0.01 ^c^	0.25 ± 0.01 ^b^	0.57 ± 0.02 ^a^
Galactinol	0.09 ± 0.01 ^b^	0.26 ± 0.01 ^a^	0.16 ± 0.02 ^b^
NN4	0.09 ± 0.01 ^b^	0.07 ± 0.01 ^b^	0.16 ± 0.02 ^a^
NN5	Nd *	Nd *	Nd *
Total cyclitols	19.29 ± 0.86 ^a^	18.95 ± 1.04 ^a^	15.20 ± 0.20 ^b^
Total galactosyl cyclitols	0.55 ± 0.03 ^b^	1.36 ± 0.05 ^a^	1.42 ± 0.05 ^a^
Cotyledons			
Maltose	0.31 ± 0.17 ^a^	0.15 ± 0.01 ^a^	0.40 ± 0.11 ^a^
Galacto-Pinitol A	0.38 ± 0.10 ^a^	0.23 ± 0.03 ^a^	Nd *
Galacto-Pinitol B	Nd *	0.09 ± 0.01	Nd *
Fagopyritol A1	0.13 ± 0.01 ^a^	0.13 ± 0.01 ^a^	Nd *
Fagopyritol A2	0.73 ± 0.15 ^a^	0.66 ± 0.17 ^a^	0.81 ± 0.08 ^a^
Fagopyritol B1	0.05 ± 0.02 ^a^	0.05 ± 0.01 ^a^	0.07 ± 0.01 ^a^
Galactinol	Nd *	Nd *	Nd *
NN4	Nd *	0.31 ± 0.01 ^a^	0.27 ± 0.02 ^a^
NN5	1.13 ± 0.13 ^a^	1.55 ± 0.53 ^a^	1.47 ± 0.13 ^a^
Total cyclitols	2.44 ± 0.03 ^b^	3.02 ± 0.12 ^a^	2.83 ± 0.27 ^ab^
Total galactosyl cyclitols	1.30 ± 0.29 ^a^	1.27 ± 0.43 ^a^	0.89 ± 0.08 ^a^

* Nd—not detected or below the limit of determination (0.05 mg/g DW). Different letters indicate significant differences between treatments at *p* ≤ 0.05 (Tukey’s post hoc test). NN4, NN5—unknown saccharides.

## Data Availability

All the data of this study are included in this article and the Supplementary Materials.

## Supplementary Materials

The following supporting information can be downloaded at: https://www.mdpi.com/article/10.3390/metabo13050672/s1, Figure S1: Chemical structures of cyclitols and their α-galactosides found in buckwheat tissues. (1) *Myo*-inositol: *cis*-1,2,3,5-*trans*-4,6-cyclohexanehexol; (2) d-pinitol (1d-3-*O*-methyl-d-*chiro*-inositol); (3) d-*chiro*-inositol: *cis*-1,2,4-*trans*-4,6-cyclohexanehexol; (4) Fagopyritol A1: *O*-α-d-galactopyranosyl-(1→3)-1 d-*chiro*-inositol; (5) Fagopyritol B1: *O*-α-d-galactopyranosyl-(1→2)-1d-*chiro*-inositol; (6) Fagopyritol A2: *O*-α-d-galactopyranosyl-(1→6)-*O*-α-d-galacto-pyranosyl-(1→3)-1d-*chiro*-inositol; (7) Galactopinitol A (*O*-α-d-galacto-pyranosyl-(1→2)-4-*O*-methyl-d-*chiro*-inositol); (8) Galactopinitol B (*O*-α-d-galacto-pyranosyl-(1→3)-3-*O*-methyl-d-*chiro*-inositol); and (9) Galactinol: *O*-α-d-galactopyranosyl-(1→1)-l-myo-inositol. Figure S2: Part of saccharide chromatograms of roots (A), hypocotyl (B), and cotyledons (C). Description of peaks: 7—NN1; 8—NN2; 9—Sucrose; 10—Maltose; 11—Galacto-pinitol A; 12—Galactopinitol B; 13—Fagopyritol A1; 14-Fagopyritol B1; 15—NN3; 16—Galactinol; 17—Raffinose; 18—NN4; 19—Fagopyritol A2; 20—NN5; and 21—Stachyose. Table S1: Values of fresh weight, dry weight, and organ length in buckwheat seedlings before and after cold stress and dehydration (mean ± SD).
