# Supplementary material for: Changes in the Carbohydrate Profile in Common Buckwheat (Fagopyrum esculentum Moench) Seedlings Induced by Cold Stress and Dehydration
Source: Metabolites. 2023 May 19;13(5):672. doi: 10.3390/metabo13050672 (PMC10223198; doi:10.3390/metabo13050672)
Supplement: Supplementary file 1 [file metabolites-13-00672-s001.zip › metabolites-2373467-supplementary.pdf]

Changes in the Carbohydrate Profile in Common Buckwheat (Fagopyrum esculentum Moench) Seedlings Induced by Cold Stress and Dehydration

Lesław B. Lahuta, Ryszard J. Górecki, Joanna Szablińska-Piernik and Marcin Horbowicz

Department of Plant Physiology, Genetics and Biotechnology, University of Warmia and Mazury, Oczapowskiego 1a, 10-719 Olsztyn, Poland

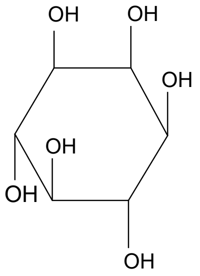

(1)

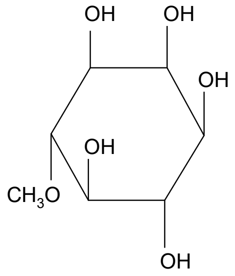

(2)

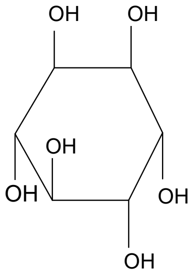

(3)

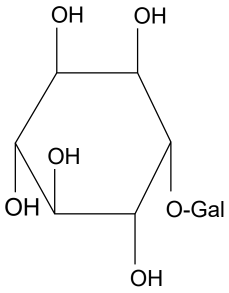

(4)

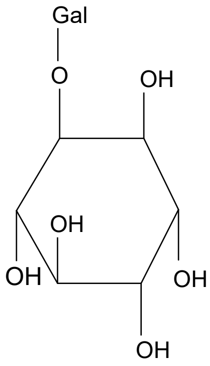

(5)

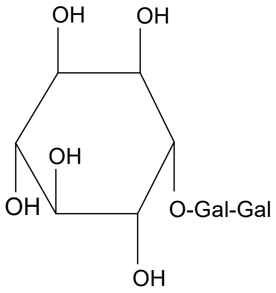

(6)

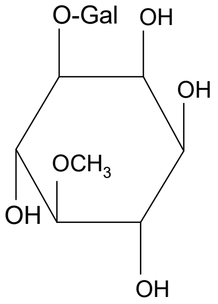

(7)

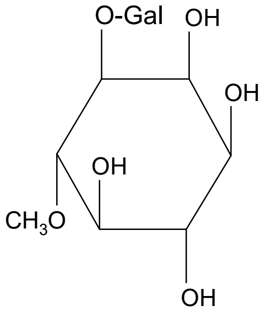

(8)

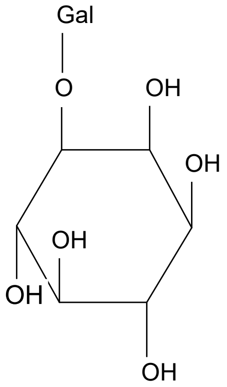

(9)

Figure S1. Chemical structures of cyclitols and their α-galactosides found in buckwheat tissues. (1) *Myo*-inositol: *cis*-1,2,3,5-*trans*-4,6-cyclohexanehexol; (2) D-pinitol (1D-3-*O*-methyl-D-*chiro*-inositol); (3) D-*chiro*-inositol: *cis*-1,2,4-*trans*-4,6-cyclohexanehexol; (4) Fagopyritol A1 : *O*-α-D-galactopyranosyl-(1→3)-1 D-*chiro*-inositol; (5) Fagopyritol B1: *O*-α-D-galactopyranosyl-(1→2)-1D-*chiro*-inositol; (6) Fagopyritol A2: *O*-α-D-galactopyranosyl-(1→6)-*O*-α-D-galactopyranosyl-(1→3)-1D-*chiro*-inositol; (7) Galactopinitol A (*O*-α-D-galacto-pyranosyl-(1→2)-4-*O*-methyl-D-*chiro*-inositol); (8) Galactopinitol B (*O*-α-D-galacto-pyranosyl-(1→3)-3-*O*-methyl-D-*chiro*-inositol); (9) Galactinol: *O*-α-D-galactopyranosyl-(1→1)-L-myo-inositol.

A. **Roots**

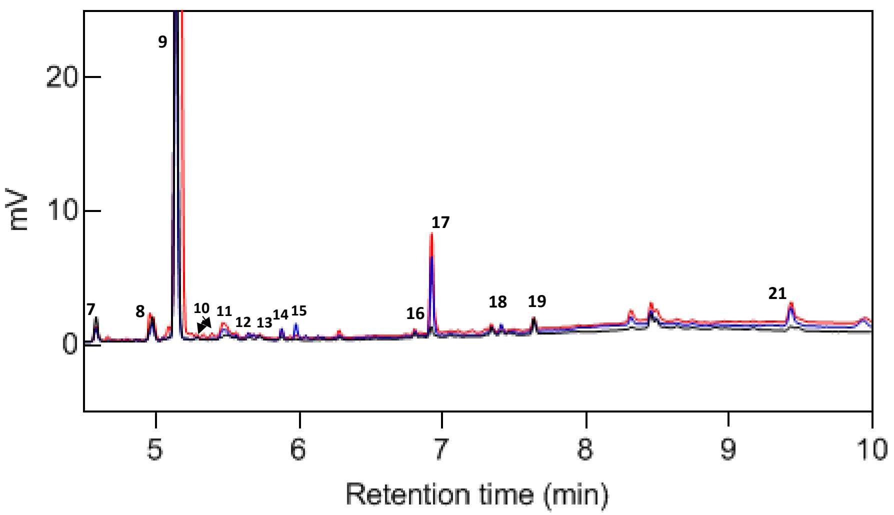

B. Hypocotyl

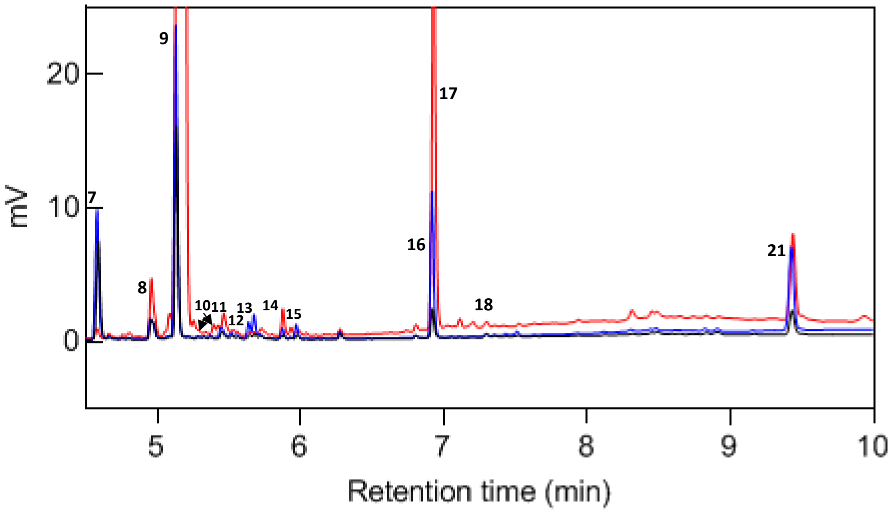

C. Cotyledons

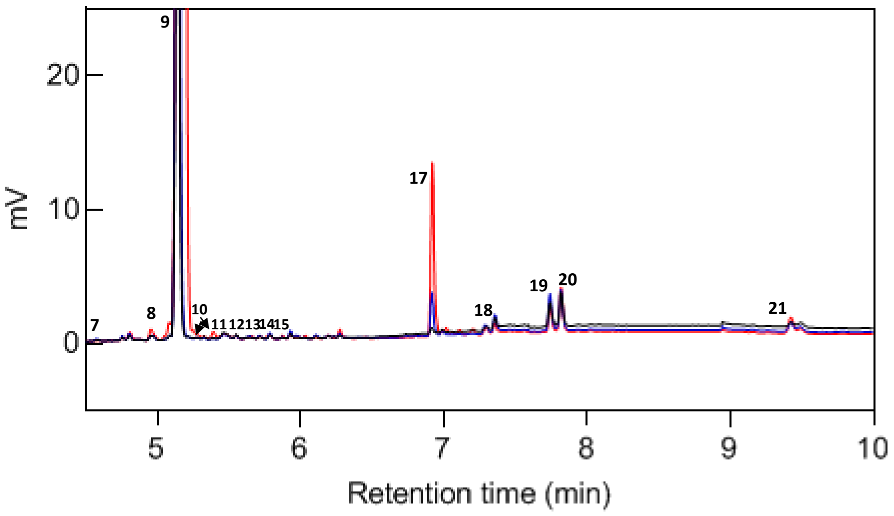

Figure S2. Part of saccharide chromatograms of roots (A), hypocotyl (B) and cotyledons (C). Description of peaks: 7 – NN1; 8 – NN2; 9 – Sucrose; 10 – Maltose; 11 – Galacto-pinitol A; 12 - Galactopinitol B; 13 – Fagopyritol A1; 14- Fagopyritol B1; 15 – NN3; 16 –

Galactinol; 17 – Raffinose; 18 – NN4; 19 – Fagopyritol A2; 20 – NN5; 21 – Stachyose. NN1 - NN5 - means not named (not known). Black line – before cold stress or dehydration; Red line – after dehydration; Blue line – after cold stress

Table S1. Values of fresh weight, dry weight and organ length in buckwheat seedlings before and after cold stress and dehydration (mean ± SD)

	**Before experiment**	**After cold stress**	**After dehydration**
Fresh weight (mg/one seedling)			
Roots	20.95 ± 1.75	20.98 ± 2.33	1.80 ± 0.11
Hypocotyl	22.24 ± 0.72	35.26 ± 1.65	1.75 ± 0.12
Cotyledons	13.64 ± 0.05	13.81 ± 0.18	4.04 ± 0.47
Total	56.83 ± 2.40	70.05 ± 3.55	7.59 ± 0.65
Dry weight (mg/one seedling)			
Roots	1.69 ± 0.13	1.61 ± 0.10	1.69 ± 0.08
Hypocotyl	2.27 ± 0.11	3.44 ± 0.02	1.69 ± 0.12
Cotyledons	4.15 ± 0.16	3.26 ± 0.28	3.84 ± 0.45
Total	8.11 ± 0.45	8.31 ± 0.35	7.22 ± 0.55
Dry weight (%)			
Roots	8.07 ± 0.07	7.70 ± 0.39	93.8 ± 0.82
Hypocotyl	10.20 ± 0.21	9.75 ± 0.39	96.5 ± 0.12
Cotyledons	30.41 ± 0.32	23.58 ± 1.67	95.2 ± 0.05
Length (mm)			
Roots	76.8 ± 2.5	76.7 ± 2.7	-
Hypocotyl	16.5 ± 0.5	22.7 ± 1.0	-
